# Active Ageing across the Life Course: Towards a Comprehensive Approach to Prevention

**DOI:** 10.1155/2021/6650414

**Published:** 2021-02-03

**Authors:** Liam Foster, Alan Walker

**Affiliations:** Department of Sociological Studies, University of Sheffield, Elmfield Building, Northumberland Road, Sheffield S10 2TU, UK

## Abstract

“Active ageing” has become the leading scientific and policy conceptualization of a later life over the past two decades in the European Union (EU). It has been used as a key strategy for responding to demographic ageing. In the United States, in contrast, discourses around successful ageing have been more prevalent. This review article charts the development of active ageing responses to demographic change, showing how the concept compares with the notion of successful ageing and other terms associated with “ageing well.” It identifies how, in practice, active ageing has been dominated by a narrow economic or productivist interpretation that prioritizes the extension of working life (to reduce the “burden” of population ageing). Such interpretations of active ageing undermine its value and emphasize the need for a more comprehensive approach which is set out. The development of the Active Ageing Index in 2012 provided a new analytical tool to promote evidence-based strategies towards population ageing. However, in practice, we show how it has not yet engaged fully with a comprehensive approach to active ageing or with the critical role of the life course in shaping the experience of old age. Nonetheless, this review article shows that the concept of active ageing still has an important role to play in our understanding of and responses to population ageing.

## 1. Introduction

Increasing longevity has led to a greater focus on the nature of later life, including how to sustain activity and health and enhance well-being [1, 2]. It has also led to considerable debate regarding the operation of welfare systems and their sustainability and, in particular, pension schemes, health care, and long-term care systems [3]. Discourses which describe the process of population ageing as a crisis or as representing a “demographic time bomb” are omnipresent in Western industrialized countries [4]. While European life expectancies are among the highest in the world, increases in healthy life expectancy (HLE) among those aged 65 to 85 tend to lag behind increases in life expectancy (LE) in the EU25, with health improvements occurring disproportionately at younger ages for men and women. In the UK, it is apparent that lower HLE is linked particularly to low occupational and income status and area deprivation [5].

The crisis rhetoric on demographic ageing and welfare provision is perceived as a threat to intergenerational solidarity [4, 6]. In effect, the notion of “earned retirement” associated with a period of leisure is, to an extent, being challenged by a moral-economic imperative for older people to stay productively engaged (primarily by extending participation in the labour market) and contributing to society [7]. This perspective has been strongly embedded in policy approaches aimed at addressing the demographic challenges.

Over recent decades, “active ageing” has become the most prominent scientific and policy approach for responding to demographic ageing in the EU [8, 9]. This has been seen as an approach to “re-negotiating the meaning and duty of old age” ([4], 94). It is this concept and its use which is the main focus of this review article. The aim is to explore the definition of active ageing, how it has been operationalised as well as how it differs from other conceptions of “ageing well,” and successful ageing in particular. In doing so, we show that despite some criticisms of the term and its use [10, 11], that when active ageing is operationalised appropriately, it still represents a valuable tool for considering ageing and how to optimise it.

Initially, the review outlines the key role of the life course in understanding the process of ageing. It then defines approaches to “ageing well,” including successful ageing, before focusing on the concept of active ageing. It considers how active ageing has been employed by policy-makers in Europe and in particular the UK showing how, in practice, it has been synonymous with a narrow employment-focused productivist approach, which mainly emphasizes working longer. It then outlines what could constitute a comprehensive approach to active ageing which, it is argued, should be central to policy strategies throughout the life course aimed at sustaining and enhancing physical and mental health and preventing multimorbidity in later life. Following this, it cross-examines the Active Ageing Index (AAI), an analytical tool to enable policy-makers to devise evidence-based ageing strategies, highlighting how it has yet to engage with a comprehensive approach to active ageing and the importance of the life course in shaping older age. It concludes that despite changes to our understanding of ageing, there is still much to do to promote a more inclusive approach to active ageing.

## 2. Ageing and the Life Course

The life course perspective powerfully shapes the process of ageing and shows how ageing is not just biologically but also socially constructed [12, 13]. It challenges static notions of “natural stages of life,” which emphasize standard age-related roles and activities [14], advocating the dynamic nature of ageing. This idea of a standardized life course has served to homogenise people into age-based categories, which are largely defined by a historically-based temporal narrative of development (and decline) [6, 15]. For example, it was only in the late nineteenth century that the “invention” of youth as a life “stage” emerged, defined in accordance with education, and symbolically perceived as the “future of the nation” [6]. In the early twentieth century, old age as a “stage” became a key component of the standardized life course through the institution of retirement and introduction of age-based pension provision. Retirement became a clearly defined “normative” stage of the life course separated from paid work that socially constructed older people as a distinct group [14, 16]. This detachment from remunerated employment can marginalise older people as “unproductive” members of society [1, 17].

In recent years, there has been a paradigm shift in the understanding of the life course, with a more fluid interpretation instead of deterministic “stages.” For example, the transitions associated with education, paid employment, retirement, and family have become increasingly blurred. The boundary between work and retirement is being redefined as a result of changing patterns of exit from paid employment and the abolition of the retirement age in some countries [18]. These have challenged the social meaning of older age and led to the notion of the “de-standardization of the life course” [14]. Pickard [6] asserts that beneath this greater fluidity lies a new structure, characterised by neoliberalism, which emphasizes individual productivity, responsibility, and success expected at all stages and in all contexts, including ill-health, unemployment, and retirement. This is hugely problematic given wide inequalities in life course experiences and circumstances in older age [10]. This is despite inequalities and individual diversity tending to increase with age [9].

The life course perspective is also valuable in understanding the role of intrinsic (genetic) and extrinsic (environmental) factors in the ageing process. The biogerontological view of ageing is that it is the consequence of cumulative wear and tear on the body [19]. However, nongenetic factors have a much greater impact on the ageing process than genetic ones [20]. For example, Steves et al. [21] found in studies of monozygous twins that inherited genes only contributed to 20% of the variance in longevity. Walker [19] argues that the key extrinsic risk factors in adulthood are associated with unhealthy practices and structure, influenced by the interactions between socioenvironmental conditions and personal and behavioural events. At an individual level, ageing does not represent a random phenomenon, despite commercial, social, and economic pressures towards unhealthy lifestyles, individuals are to some extent agents of their own ageing process. For instance, unhealthy practices, including poor diets, smoking, lack of exercise, and alcohol use are widely acknowledged to negatively influence LE and HLE. More importantly, structural risk factors, including social exclusion, deprivation, and low socioeconomic status have a huge direct impact on ageing, including cognition [22], as well as framing health practices. For example, financial pressures associated with low income can raise stress, increase blood pressure and depression, and reduce access to preventative health measures that incur costs. This can result in chronic conditions, which are associated with “loss of function in later life, or biological ageing” [19].

Given that none of these biological and environmental connections take place exclusively in older age, the life course is crucial to our understanding of ageing [23]. This is why social policy measures aimed at improving well-being in later life, including active ageing, must adopt a life-course approach. For example, action to limit risk factors in early and midlife is likely to improve physical and mental health in older age.

## 3. Ageing “Well”

A plethora of terms are used to express “ageing well,” including, healthy ageing, positive ageing, productive ageing, successful ageing, and active ageing. These concepts each imply (sometimes subtly) different approaches to the possibilities presented by ageing [24, 25]. They also differ in their portrayal of the role of older people and the extent to which they incorporate a life course perspective. For instance, some theories of ageing linked to a decline and loss paradigm associated with “normal ageing” have employed static interpretations of the life course, prioritizing biology [26]; while others, such as comprehensive interpretations of active ageing, emphasize the centrality of the life course [8]. What they have in common, however, is that they are multidimensional and multilevel concepts which represent a new paradigm in gerontology based on the compression of morbidity and mortality, the delay of senescence, and, in theory, recognize wide diversity in the ageing process [25, 27].

Active ageing has become the most widespread term employed in European research and policy discourses over recent decades, while successful ageing has dominated in the USA. Although used interchangeably [26], active ageing and successful ageing have inherent differences. Successful ageing is a multidimensional measure which was initially developed as a tool for conducting research to establish “an intellectual and methodological foundation” for a new gerontology [28], as opposed to an idea to be employed in policy formulation (although it has subsequently been used for this purpose), whereas active ageing was designed explicitly for policy-making [29, 30]. The focus of active ageing, in the WHO's [8] widely used formulation, is on mainstreaming participation and health over the life-course. Although the WHO's [31] most recent strategy is built on a conceptualisation of healthy ageing outlined in the “World Report on Ageing and Health,” like active ageing, it embraces a life course approach to health which recognizes the impact that early life experiences have on ageing. It also aims to maximize older adults' physical, social, and mental well-being in order to promote independence and reduce the burden on others [32, 33]. Furthermore, both concepts emphasize the need for action across multiple sectors while enabling older people to remain a resource to their families, communities, and economies. There are numerous examples of the coexistence of the terms in documentation referring to “ageing well,” including in relation to the AAI, which is defined as an “active and healthy ageing measure” [2]. However, there are also concerns that the definition of healthy ageing could be interpreted in a manner which places too greater emphasis on functional ability (see [34] for a discussion).

In the US, over the last four decades, successful ageing has become the prominent discourse concerning growing older and has been characterised by the rejection of older age as an inevitable succession of losses [1]. This has led to a change in focus from those “doing poorly to those doing well” [35]. It aimed to eclipse previous thinking which focused on disengagement and unavoidable senescence, by concentrating on activity and function [36]. Moreover, the model solidified a major turning point in gerontology, with research targeted on those who have positive experiences of ageing rather than those who suffer illness and disabilities as they age [37]. This view of ageing has represented a positive step in acknowledging the varied contribution older people play in society, but it has also served to exclude those considered to be “doing poorly” due to their lack of “success.”

Both successful and active ageing are directly linked to the activity perspective that emerged in the early 1960s, as the antithesis of disengagement theory. Cumming and Henry [38] assumed disengagement was a universal and inevitable process and outlined the “natural” tendency to disengage from society (and work) as people aged. By removing responsibilities deemed to be increasingly burdensome, older individuals were provided with space to confront their own impending death [29]. Disengagement theory provided a functionalist account of old age as a distinct stage in adult development, part of a normative life course. The theory was criticised for neglecting older adults' own perceptions regarding what engagement constituted, the heterogeneities in how individuals experience later life, and for enforcing a deficit model [39]. In contrast, successful ageing contends that to age well it is important for individuals to maintain mental and physical capacities to enable continued social activity in older age [40]. In effect, middle age was to be extended and older age denied [41].

First coined over 40 years ago by Butler [42], successful ageing increased in popularity following an article by Rowe and Kahn [43] where it was argued that ageing and illness are distinct processes. Rowe and Kahn [44] elaborated on their initial model of successful ageing to include three main components: low probability of disease and disease-related disability, high cognitive and physical functional capacity, and active engagement with life. This model has contributed to the rejection of the perception that older age is inextricably linked to an unavoidable series of losses. In doing so, it served as a preventive and optimistic approach to later life [45]. It evoked new narrative of positive ageing, emphasizing self-directed health across the life course. It indicated that experiences in later life could be considered in terms of success, rather than in conventional expectations for failure. While many different versions of successful ageing have subsequently emerged, Rowe and Kahn's model is still the most widely referenced [28, 46].

However, this version of successful ageing has a number of limitations. For example, it prioritizes physical and mental capabilities over social and behavioural aspects (engagement with life). Rowe and Kahn overestimated the number of older people progressing disease-free through older age. Evidence shows that ill-health and disability are common in older age [5, 22], pointing towards the unattainability of successful ageing according to Rowe and Kahn's criteria [25] for many older adults [1, 47]. Furthermore, a significant number of the oldest-old who do not comply with Rowe and Kahn's strictures still exhibit considerable levels of psychological well-being [23, 48]. “Successful” implies that there are winners and losers in the ageing process. However, most gerontologists are uneasy about the prospect of labelling someone as unsuccessful as a result of disability or ill-health [35].

Successful ageing is often concerned with how older individuals should age, not how people view themselves as ageing successfully [49, 50]. In effect, this binary interpretation of successful ageing results in an oversimplification, which serves to conceal that people can be content and high functioning despite the absence of dimensions of Rowe and Kahn's model [23, 51, 52]. There is a risk that successful ageing is reduced to an exclusionary and even discriminatory perspective.

Furthermore, successful ageing is an individualistic concept which fails to incorporate the fact that variations in peoples' lives and social structural position are interdependent [53]. It assumes that “through individual choice and effort” people can age successfully and remain physically and socially active ([44], 37). By aligning to the role of individual volition and lifestyle in relation to health, Rowe and Kahn moved successful ageing further from a social determinants perspective [46], failing to recognize the impact of structural impediments throughout the life course [4]. Riley ([54], 151) labelled the model “seriously incomplete” because of its neglect of these structural factors that impinge on ageing and its sole focus on individual success. By emphasizing the responsibility of individuals to sustain physical and cognitive function, it could reinforce attempts to limit state responsibility to provide needed resources, both in older age and to address social and structural inequities throughout the life course [33, 55]. As such, it has become rooted in neoliberal ideals of personal autonomy and responsibility [56]. Furthermore, by focusing on late adulthood, Rowe and Kahn's model makes a static assessment of an individual's successful ageing, failing to engage with a life course perspective and the developmental processes and trajectories of continuity and change in function [37]. Thus, it fails to capture developmental processes of continuity and change in function over time.

These concerns have led to the emergence of other definitions of successful ageing. For example, Baltes' psychology based selection-optimization-compensation (SOC) model [57] and Carstensen's [58] socioemotional selectivity theory. These more life-course oriented approaches emphasize the “how” of successful ageing rather than focusing on the “what.” Bodily decline and reduced plasticity over the life course were seen as a premise for interventions to promote successful ageing [28]. While there is no disagreement regarding the requirement to optimise and empower individual to achieve “success,” the criteria employed for defining success differs [46]. Operational definitions have tended to be based on objective measurements of health and functionality, failing to take into account individual's perceptions of their own experiences of health and wellbeing, which would enable a more comprehensive view of ageing [25]. For instance, Kleinedam et al. [59] call for a well-constructed approach which includes measurements of physiological health, well-being, and social engagement, with subjective and objective aspects. This subjective element would provide greater attention to individuals' perceptions of their own ageing and the effects of earlier life experiences. Other scholars' state that disability should be included in the conceptualisations [46]. This all indicates the need for a universal description and consensus of what successful ageing and its operation entail [25].

## 4. Active Ageing

Active ageing developed in the 1990s with a distinct emphasis on the relationship between health and activity [60]. Its emergence corresponded with the dismantling of the traditional conception of the life course which associated the oldest “stage” of life with inactivity [61]. The most extensively used definition of active ageing is from the WHO: “the process of optimizing opportunities for health, participation and security in order to enhance quality of life as people age.” “Active” was defined as “continuing participation in social, economic, cultural, spiritual and civic affairs, not just the ability to be physically active or to participate in the labour force” ([8], 12). This definition, similarly to successful ageing, challenged stereotypes of older age focused on dependency and passivity, emphasizing autonomy, and participation [62]. In doing so, it refutes the “decline and loss paradigm” often linked to the effects of physical decrescence [15] and highlights the active roles older people play in society.

Active ageing indicates the important distinction between activity and passivity, but promotes the notion of being active as involving living by one's own rules rather than those “normalized” by others [63]. It also requires activities that are aimed at ensuring the protection, dignity, and care of people as they age [64]. It represents a view of the possibilities of ageing not purely in economic terms, but in a more holistic manner, including social participation, and mental and physical well-being [65]. It also highlights the importance of earlier life course events in determining well-being in later life [66], underlining the need for prevention. This includes providing opportunities to contribute to pensions or rewarding periods of caring in order to prevent poverty in older age [67], and health promotion measures to limit ill-health in later life [19]. In older age, active ageing promotes opportunities to participate in society, including paid employment. This requires measures to combat age discrimination and promote age diversity, training, and flexible forms of employment [18].

Active ageing is not without criticism. It has been argued that an idealization of it could be counterproductive and oppressive [47] and that policy-makers overemphasize physical activity while neglecting mental capacity, and too often equate it with working longer [9, 68]. Tornstam ([69], 322) criticizes middle-aged academics and policy-makers who have a tendency to apply their own standards to those of older people, even though they may not accurately depict the priorities of older people. He points to “an overflow of mid-life values found in society at large […], which means that our choice of conceptual delineations and theories carries the (sometimes hidden) stamp of values that emphasize productivity, effectiveness, and independence.” Consequently, there have been criticisms of conceptions of active ageing for promoting biased policies which, in effect, privilege or impose particular lifestyles [10, 11]. Even those who promote active ageing are aware of the risk “that this sort of strategy will become coercive” [41]. Thus, it could serve to contribute to the exclusion of the oldest-old, and those most vulnerable and dependent, who fail to meet inappropriate active ageing criteria [1, 70]. Pfaller and Schweda ([10]: 47) state that where active ageing is operationalised in a manner which emphasizes personal responsibility, similarly to successful ageing, “it actually functions as a mere alibi for dismantling the welfare state and shifting risks and costs to the single individual.” This necessitates older people's close involvement in determining what role active ageing could play in their lives, including involving their involvement in the coproduction of policies [62]. By contributing their own understandings of the issue, it enhances the prospect of producing findings of relevance for the well-being of older people [71].

## 5. Active Ageing in European Policy Discourses

Since the 1990s, the Active Ageing paradigm gradually gained ground in European institutional, professional, and scientific spheres as “the ideal framework for public policy planning and for responding to the population's ageing” ([9], 406). Ageing first came to prominence as an important European policy issue in the early 1990s. The European Year of Older People in 1993 advocated a new participative discourse on ageing. The policy document, “Towards a Europe for all Ages” [72], led the European Commission (EC) to four policy conclusions: to raise employment rates of older people in Europe (through promoting lifelong learning, flexible working arrangements and incentives), to reverse trends in early exit from the labour market and improve social protection policies, to support health policies and old-age care research, and to develop policies to combat workplace-based discrimination and social exclusion. Despite the extensive scope of active ageing policy, it was subsequently narrowed with employment becoming the primary focus [17], although they also make reference to health interventions, for example, through the maintenance of healthy lifestyles [9]. Even policies associated with a more comprehensive approach to ageing are often underpinned by productivist aims. For example, policy documents in the UK have prioritized the role of education in advancing employment in older age, as opposed to focusing on opportunities for personal development [73]. The EC [74] “Ageing Report” emphasized a productivist approach to ageing, stating that raising the retirement age, limiting early retirement, and greater links between pension contributions and benefits would incentivise continued labour market participation.

This productivist vision, in accordance with a neoliberal individualisation of responsibilities, advocated the need for “activated” older workers to enhance economic growth [66]. This was apparent in the so-called Lisbon target to raise the employment rate of those aged 55-64 to 50% by 2010, which only 11 EU countries managed to achieve [75]. This model assumes the availability of paid employment which is suitable and fails to address the effect of structural inequities [18, 53]. Retirement can be transformed from an expectation or reward for individuals' “productive” years to an undesirable status more associated with a lack of employment [76]. Thus, there is a danger that active ageing policy becomes synonymous with work. This could result in a new form of ageism, which requires continuation of work as the “new legitimacy for a mature identity” ([77], 254).

This is not to ignore that some EU documentation considers a broader range of measures, such as lifelong learning, health-promoting activities, and activity after retirement. For instance, a year after the Ageing Report, a more comprehensive approach was highlighted by the EU Council ([78], 5):

“Active ageing means creating opportunities for staying in the labour market longer, for contributing to society through unpaid work in the community as volunteers or passing on their skills to younger people, and in their extended families, and for living autonomously and in dignity for as much and as long as possible”.

More recently, the EC [79] has defined active ageing as “helping people stay in charge of their own lives for as long as possible as they age, and, where possible, to contribute to the economy and society.” Thus, there have been two contrasting EU policy discourses on active ageing over recent decades, but the most prevalent one has been productivist, focusing on the extension of working lives. Alongside it is a more comprehensive approach to active ageing along the lines promoted by the WHO, which focuses on participation in a broad sense, increasingly emphasizing the importance of prevention throughout the life course on health improvements in later life [9]. Despite some evidence of convergence, in practice, policy instruments still focus primarily on paid employment [16]. This is linked to the financial implications of population ageing, pointing to the importance of productive activities in addition to maintaining independent and healthy lifestyles in order to counter age discrimination and extend employment [9].

## 6. Active Ageing at a National Level: The UK

The UK's responses to the challenges of ageing have tended to largely focus on productivist notions of active ageing, with other more comprehensive responses tending to be reactive and largely remedial [19]. This is despite some promising signs in the early years of the century [80–82]. For instance, the DoH's [81] National Service Framework for Older People represented an attempt to extend the healthy life expectancy of older people, incorporating a national standard that “the health and well-being of older people is promoted through a coordinated programme of action led by the NHS with support from councils” (14). It emphasized the need for older people to be able to access health promotion activities (including factors such as smoking cessation) as well as the benefits of a wider range of initiatives associated with health and wellbeing, such as tackling poverty through benefits advice and support [32]. However, a decade later, these measures to promote healthy life expectancy at age 65 remained a tier 3 priority, meaning that primary care trusts could choose to prioritize it locally, or not [83]. More recently, a Foresight Review [84] considered some of the main policy questions arising from population ageing, including longer working lives, housing, health, and the family, but failed to adopt a comprehensive framework.

One of the major challenges confronting a more comprehensive approach to active ageing in the UK is that it requires a collective approach in order to mobilise a wide range of societal resources, underpinned by a commitment to public welfare. This collectivism is highly problematic under neoliberal policies such as in the UK, where individual responsibility is designated to play a substantial role [85]. This goes some way to explain why successive UK governments have thus far failed to introduce far-reaching public health reforms for instance. In addition, any commitment to wide-ranging and preventative welfare provision will be severely tested by the difficult financial climate linked to Covid-19. Walker ([19], 269) states that “it is not that successive governments have openly opposed a life course approach to ageing but, rather, if they have even contemplated it, the ideological and/or practical challenges of doing so have proved too daunting.” In practice in the UK, a life-course focus, which is a key component of active ageing, is often overlooked with old age being spotlighted instead [32]. Therefore, a comprehensive approach to implementing ageing policy requires a substantial ideological shift in the UK.

In contrast, productivist policies associated with active ageing, often characterised by a focus on individual responsibility, have tended to flourish in the UK [3]. In accordance with the recommendations of the EC's [74], “Ageing Report” measures have been implemented to raise the retirement age, restrict access to early retirement, and provide a stronger link between pension benefits and pension contributions, in effect, creating incentives to remain in the labour market. For instance, in 2010, the default retirement age (DRA) of 65, which meant employers could force their employees to retire at the age of 65, was abolished (a few employers such as the fire service still have a compulsory retirement age). This was seen as a useful way of “encouraging” people with inadequate retirement incomes to continue working and contributing to pensions [18]. In the UK, pension policy has encouraged delayed retirement in a number of ways, including increases in the age at which the state pension can be received (the 2011 Pension Act will result in a phased increase in the SPA to 68 between 2037 and 2039) (see [86]). Furthermore, the UK is unusual by international standards in significantly raising the State Pension Age (SPA) without enabling people to take a reduced pension if they leave paid employment prior to it [18]. These productivist active ageing strategies have been justified by the need to raise employment levels in the context of ageing populations and projected increases in pension costs [3].

The Department for Work and Pensions (DWP) ([87], 5) has argued that “early exit from the labour market can have serious implications for the health, well-being and incomes of individuals, and comes at a significant cost to the economy, business and society as a whole.” The danger here is that those not in paid employment are excluded from ageing actively, risking devaluing the valuable contributions they make to society [1]. This rather utilitarian vision serves to promote the responsibility of older workers to be “activated.” In practice, recent narratives of working longer are imbued with a notion of obligation on the part of the older worker who should have a duty to avoid becoming a “burden” on society [77].

## 7. A Comprehensive Approach to Active Ageing

There is still little consensus among the different experts and institutions about a precise operational definition of active ageing [9]. Therefore, in order to maximize the scope of active ageing and its prospective impact on ageing, as well as addressing the criticisms of this paradigm, it is important to identify what a comprehensive strategy might constitute, including its underlying principles. Building on and expanding the WHO [8] definition, Foster and Walker [16] proposed a series of key principles as the basis for a comprehensive strategy on active ageing. We suggest that these represent a useful starting point for embracing a comprehensive approach to active ageing and its operationalization.

First, “activity” should embrace all meaningful pursuits which contribute to individual well-being. This means activities such as volunteering and caring should be as valued as paid employment [1, 26], a principle not evident in the AAI [88]. There is also considerable evidence that increased engagement in social and leisure activities has the potential to improve (physical, cognitive, and emotional) health and well-being in later life [61, 70]. This may be through social support, reduced stress, persuasion and support, social interactions, and a reduction in social isolation [89]. Age Platform Europe ([90], 9) has concluded that “isolation, invisibility and loneliness are important issues for many older people that hinder their integration into society and undermine the aim of active and healthy ageing.” In addition, increased participation has the potential to reduce expenditure on health care provision [2, 65].

Second, it should be largely preventative, aiming to include all ages in ageing actively across the life course. The life course perspective is essential here as circumstances in later life are associated with social and economic status in earlier life, and exposure to risk factors. The use of preventative health interventions to improve lifestyle, diet, and consumption patterns are important in determining health at all ages [19]. A shift from a primarily curative to a more preventive medical focus with a focus on life-long prophylaxis and prevention is central to this approach [4]. Third, active ageing should be inclusive. It should not exclude those who are frail and dependent or focus only on the “young-old,” neglecting the “old-old.” Thus far, it has been argued that active ageing policy in the EU has largely been concerned with the young-old (and employability) rather than the old-old, where the chances of experiencing cognitive and physical deficits increase substantially [57]. Fourth, intergenerational solidarity should be a central component of active ageing, involving fairness between generations. It is important to ensure that the interests of all generations (including future ones) are taken into account in the operation of pensions, health, and long-term care and that appropriate narratives of intergenerational relations are presented [6]. Hess et al. [91] found that in Germany and the UK, active ageing positively framed how older people and their contributions to society are seen, decreasing perceptions of older people as a “burden.”

Fifth, the concept should consist of both rights and obligations in relation to social protection, lifelong education, and training for instance. This does not mean sanctions should be applied for noncompliance but rather education is required about the need for personal in addition to sociopolitical responsibility early in the life course in the adoption of preventative measures [19]. Sixth, active ageing strategies should be empowering. This means both top-down policy action to assist activity and opportunities for citizens to influence action from the bottom up. This potential can be affected by financial status, health, and mobility in older age [92]. Facilitating people in older age to actively engage with society also necessitates the input of resources [26].

Seventh is the need for active ageing to respect diversity. There is a danger that active ageing could serve as “another way to oppress marginalized and disadvantaged elders” ([93], 716). Therefore, it needs to operate in a manner which is more sensitive to cultural diversity in ageing and promote social inclusion. There are large variations within (and between) countries across Europe in both activity patterns, preferences and norms [22]. Finally, a comprehensive approach to active ageing must be flexible. It must accept that as individuals age throughout the life course, this results in changing ideas of what active ageing means to different people. Alterations in preferences and constraints emerge throughout adulthood. Boudiny [1] contends that adaptability is needed, whereby active ageing policies assist people to accept changes and integrate them into their lives. This may be through the use of training interventions (not simply associated with employment) [18], environmental modifications, assistive devices [2], and ICT [63].

## 8. The Active Ageing Index (AAI)

Having set out a vision for a comprehensive approach to active ageing, it is important to consider the extent to which such an approach has been advocated in recent attempts to measure active ageing, in order to assist in the targeting of policy solutions. The European Year of Active Ageing and Solidarity Between the Generations in 2012 ageing led to the emergence of a composite quantitative measure of active and healthy ageing for EU countries called the AAI [94]. The AAI focuses largely on concepts, definitions, and social policy discourses. It provides a comparable multidimensional resource on the circumstances of EU countries in relation to active ageing [95], “to support national policymakers in designing successful responses to the challenges of population ageing” ([96], 3). It can be used to monitor societal progress relating to active ageing [29, 91] and to devise evidence-based strategies [94].

The AAI consists of 22 gender-specific outcome indicators, aggregated into domain-specific composite indices of the following four domains: (1) contributions through paid activities: employment; (2) contributions through unpaid productive activities: participation in society; (3) independent, healthy, and secure living; (4) capability to actively age: capacity and enabling environment for active ageing. There are tough questions about how the data are derived and utilised. First, the indicators come from various surveys where any dispersion across individuals will be lost when constructing national averages [95]. Second, there is the challenge of ensuring consistency among variables from different data sources, “however inadequate they might be” ([97], 157). More detailed comparative data will be needed in order to distinguish between health status, levels of education, and age cohorts, among others. Third, the AAI process of calculating the different weights for each indicator was arbitrary: by means of consensus within an AAI Expert Group. Those domains with the highest weightings are those associated with more productivist notions of active ageing [98].

Amado et al. [88] assert that the attributed weights in the AAI are fixed in structure with equal weights for each country, which assumes homogeneity, rather than the social, economic, and political reality of diversity between (and within) countries. There are concerns that the AAI proposes unobtainable and prescriptive goalposts to be achieved, which neglect differences in individual capacities, resources, and preferences, and does not accurately capture cultural diversity encouraged in a comprehensive approach to active ageing [11, 30].

While the approach used in calculating the AAI is problematic, it still provides important information on the experiences of older people for a wide group of stakeholders [2]. It has identified considerable diversity among EU countries in relation to active ageing. For instance, Nordic countries, the United Kingdom and Ireland rank highest, whereas the majority of Central European countries and Greece are located at the bottom. The gender analysis also shows that AAI scores tend to be higher for men [94]. This gender disparity is most prevalent in the first domain (employment) and also in the third domain (independent living), areas where the gender gap in financial security is extensive in many EU countries [17]. This disparity is associated with unequal employment experiences during the life course, which has considerable implications for the incomes of older women [65]. That said, it is evident that more work is required to enhance its capacity as a comprehensive policy-making tool. In particular, while outcomes in later life exhibited in the AAI enable life course policies to be framed, the AAI does not actually reflect the life course perspective inherent in active ageing [97]. Broadening the scope of the AAI could facilitate an extension of knowledge and more effective promotion of active ageing [95]. This includes greater engagement with the comprehensive active ageing approach set out above and a commitment to the provision of comparable longitudinal comparative data which can facilitate a life course approach to our understanding of ageing. Despite the arrival of the AAI as a potential policy tool, in its current form, there is still more to be done to incorporate a comprehensive approach and, especially, a life course perspective.

## 9. Conclusion

Dependency has traditionally been the starting point for discussion about old age. However, more positive notions of ageing, such as active and successful ageing, have begun to move debates beyond this deficit model. This is important in the context of attitudes towards older people in ageing societies. However, if they are narrowly applied, both risk the creation of a two-tiered view of the older population with a minority of people (predominantly the young-old) aspiring to meet standards of success or activity (linked to employment), which remain unattainable for many [47]. A strict rubric of success or activity excludes too many and fails to embrace differences between older people. While both concepts still receive considerable attention, we argue that active ageing is a broader-based concept than successful ageing based on Rowe and Kahn's definition [61] and, as such, appears to offer greater positive policy potential than other ageing discourses. However, geographical tendencies in their usage mean they are likely to continue to coexist, accompanied by other associated concepts of ageing well.

In practice, policy-makers have prioritized productivist approaches to active ageing, marginalizing those who are unable to work, or do not wish to fulfil certain roles assigned to them [98]. A comprehensive active ageing agenda eschews productivism and focuses on policies for promoting well-being as people age, which recognizes diverse needs and operates in a noncoercive and inclusive manner [2, 16]. While the AAI represents a positive attempt to provide a policy tool to monitor active ageing, it is evident that further research is required to operationalise it in a comprehensive life course context. This focus is fundamental to the effective implementation of an active ageing approach. It would reject approaches to ageing where older people are seen as a distinct homogeneous group and the outdated notion of a “normative” life-course where older age is regulated extensively by age-based criteria regarding exit from the labour market [6]. Moreover, it would recognize the role of experiences in childhood and midlife and their long-term consequences for adjustment and functioning in later life. A life-course perspective focuses on the historical, cultural, and social context for ageing [13], enabling a nuanced perspective of how individual agency and social structures interact to influence ageing outcomes [37].

The ageing process is strongly influenced by extrinsic or environmental factors, operating throughout the life course, as well as intrinsic genetic factors, including predispositions to certain health conditions [19]. “At the biological level, ageing results from the impact of the accumulation of a wide variety of molecular and cellular damage over time. … But these changes are neither linear nor consistent, and they are only loosely associated with a person's age in years” [99]. As such, chronological age does not represent an effective predictor of performance [41]. Moreover, diversity in older age is not entirely random. Relationships with environments are skewed by various personal characteristics, such as the family people are born into, sex, and ethnicity, which lead to differences in health and income in later life [6]. Therefore, active ageing policy should be seen as an intervention in the entire ageing process—from cradle to grave [98]. This reinforces the importance of a preventative dimension. For example, ageing well requires strategies to promote health and well-being prior to older age. This includes more active interventions to prevent the causes of individual loss of cognitive and physical function in later life. This includes medical support and education regarding risky behaviours and a redistribution of resources from acute to preventative health. In addition, policies which are aimed at enhancing living standards and education are likely to positively impact on ageing in the form of increased later-life cognition as well as employability [19]. Policies aimed at maintaining physical and mental capacity are likely to assist older people to work longer, in addition to creating healthier and more fulfilled postemployment years [65]. In older age, active ageing means providing opportunities to continue to participate in employment (paid and unpaid), communities, and engage in new activities.

Progressive policy proposals are of limited benefit unless they are accompanied by appropriate official actions, but this is problematic in the context of neoliberalism, which has encouraged productivist approaches to active ageing [24]. Many European countries have prioritized austerity policies since the 2010 financial crisis, including the UK, and will continue to do so in the context of Covid-19. These have served to expose many of the poorest groups in particular to additional risks [100]. This context is not conducive to implementing a comprehensive approach to active ageing, which requires a collective approach underpinned by commitment to welfare provision. The dominance of a neoliberal ideology with its promotion of anticollectivism has eroded opportunities for a life course focused active ageing agenda to promote mental well-being and reduce multimorbidity in later life. This means a fundamental ideological change is required if ever active ageing can be implemented thoroughly.

## Data Availability

The article is a review article that does not rely on empirical research.
